# The effect of food environment on nutrition-related health: evidence from rural China

**DOI:** 10.1186/s41043-025-00972-6

**Published:** 2025-07-03

**Authors:** Lixia Zhang, Shaoting Li, Laiwu Zhang, Weigang Liu

**Affiliations:** 1 College of Economics and Management, Northwest A&F University, 3 Taicheng Rd, Yangling, 712100 Shaanxi China; 2 Six Industrial Research Institute, Northwest A&F University, 3 Taicheng Rd, Yangling, 712100 Shaanxi China; 3 Department of Agricultural Economics, Kiel University, Wilhelm Seelig Platz 7, 24118 Kiel, Germany

**Keywords:** Food environment, Nutrition-related health, Overweight, CHEI, DBI, Rural residents, China

## Abstract

**Background:**

Understanding the impact of the food environment on nutrition-related health is essential for addressing the rising prevalence of issues such as overweight and obesity amid rapidly changing dietary patterns in many emerging economies. This study aimed to explore the cause relationship between the food environment, including its three sub-dimensions—food availability, accessibility, and affordability, and nutrition-related health outcomes.

**Methods:**

This study utilizes survey data collected from rural households in Shaanxi Province, China, in 2022. This analysis utilizes 2SLS and IV-Probit models to analyze the relationship between food environment and nutrition-related health outcomes.

**Results:**

The findings suggest that the food environment and its sub-dimensions significantly increase nutrition-related health among rural residents. Specially, based on supermarkets and free markets, the food environment has a significant negative impact on BMI and overweight. Besides, food availability and accessibility in rural areas based on supermarkets and free markets significantly increase the nutritional outcomes. To explore the underlying mechanisms, we further analyze the mediating roles of nutrition literacy and dietary quality, the latter of which is evaluated using the Chinese Healthy Eating Index (CHEI) and the Dietary Balance Index (DBI). The results confirm that the food environment positively influences both nutrition literacy and dietary quality.

**Conclusion:**

Enhancing the food environment is an effective pathway to improving nutrition-related health outcomes in rural China. Policymakers should prioritize dynamic improvements in food availability and accessibility—particularly through supermarkets and farmers’ markets—while also promoting nutrition literacy and diet quality to support long-term public health goals.

**Graphical abstract:**

Conceptual framework of the relationship between the food environment and nutrition-related health outcomes. 
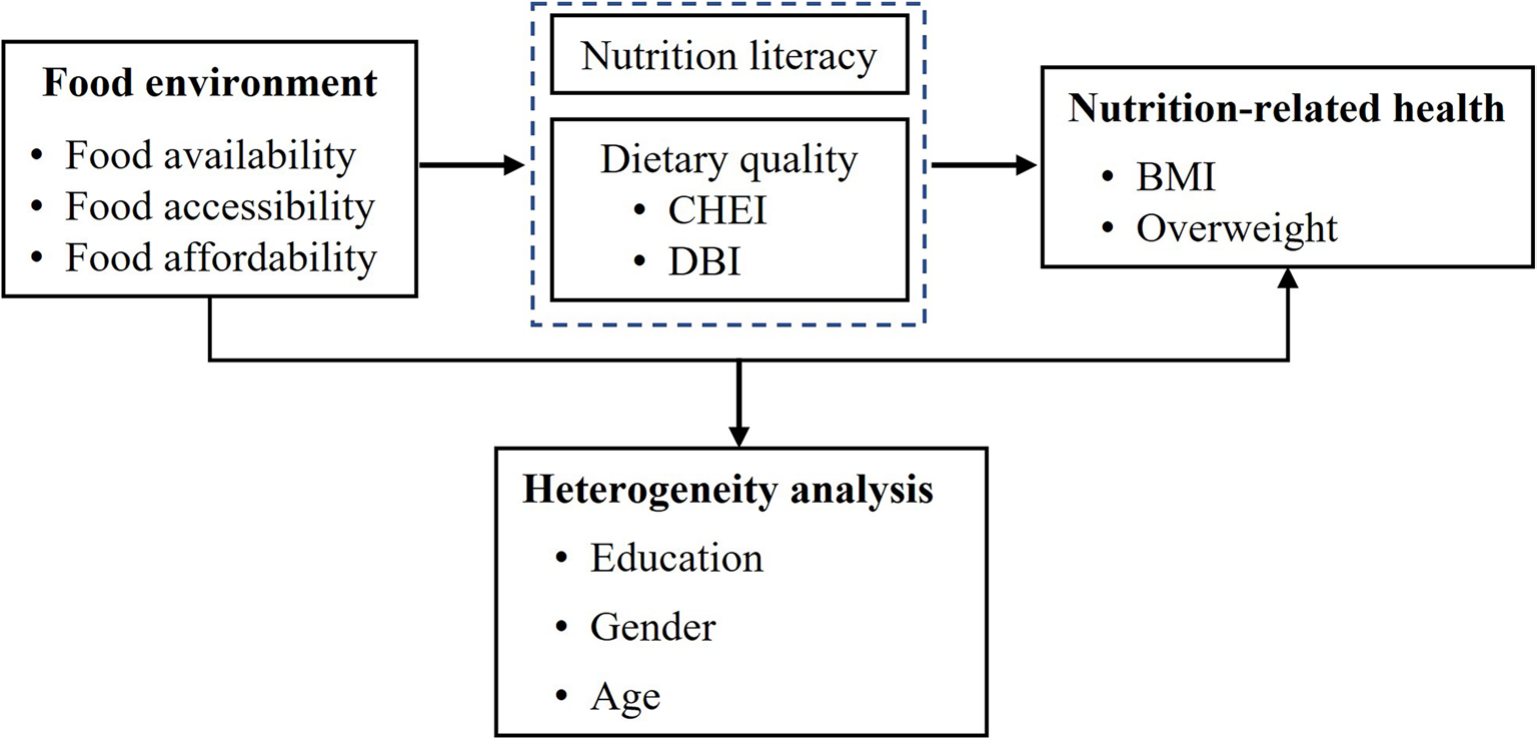

## Article highlights

1. Establishes a causal relationship between the food environment and nutrition-related health outcomes in rural residents.
2. Explores underlying mechanisms, identifying nutrition literacy and dietary quality as key mediating channels.
3. Provides valuable insights for policymakers seeking to improve public health in developing countries.

## Introduction

Many low- and middle-income countries are experiencing a rapid shift in dietary habits, marked by increased consumption of calorie-dense sweeteners, processed products, and animal-based foods. One notable consequence is the growing prevalence of overweight and obesity, which poses serious threats to public health [1]. Overweight and obesity have become major global health issues, affecting over 2.5 billion adults and contributing to chronic diseases like diabetes and cardiovascular illnesses [2]. From 1990 to 2022, obesity rates more than doubled for women (8.8–18.5%) and nearly tripled for men (4.8–14.0%) [3]. Obesity is now responsible for over 1.3 million deaths annually, making it the fourth leading risk factor for death [4, 5]. The economic impact of obesity is substantial, with rising healthcare costs and increased medical expenditures related to obesity-related conditions such as diabetes, hypertension, and cardiovascular diseases [6]. While once considered a problem exclusive to high-income countries, the prevalence of obesity is rapidly increasing in low- and middle-income nations, often coexisting with undernutrition in the same communities [7]. China exemplifies this shift. Over the past 2 decades, the country has experienced a dramatic rise in overweight and obesity rates among both adults and children. This trend is not limited to urban areas; rural communities are increasingly affected [8–10]. By 2030, an estimated 65.3% of Chinese adults (approximately 790 million) may be overweight or obese, with associated medical costs projected to reach US $61 billion, representing 22% of national health expenditures [11].

Dietary patterns and nutrition outcomes are strongly shaped by the characteristics of the surrounding food environment. Generally, food environments are defined as “the collective physical, economic, policy and socio-cultural surroundings, opportunities and conditions that influence people’s food choices and nutritional status” [12]. A growing body of evidence suggests that both the physical availability of food and the broader socioeconomic and policy contexts significantly influence individual and community-level dietary choices [13, 14]. The built food environment—part of the human-designed landscape—affects dietary quality by shaping the ease of access to, and the adequacy of, available food within a given area [15]. In low- and middle-income countries, rapid urbanization, shifts in food systems, and the widespread availability of ultra-processed foods have led to a “double burden” of malnutrition, characterized by the coexistence of undernutrition and rising obesity rates [16]. In China, these challenges are particularly pronounced due to rural–urban disparities and changing consumption patterns brought about by market-oriented reforms over the past 4 decades. As household self-production becomes insufficient to meet the rising demand for dietary diversity, rural residents are increasingly reliant on the food market, particularly in terms of food availability [7, 8, 17]. Meanwhile, the growing affordability and accessibility of ultra-processed, high-calorie foods—often more prevalent than fresh and minimally processed alternatives—have contributed to deteriorating nutrition outcomes [18, 19]. Rural areas also face structural constraints, including limited transportation infrastructure, low vehicle ownership, and underdeveloped retail systems, which reduce access to healthy food options and result in lower availability of fresh produce, dairy, and protein-rich foods [20]. These factors contribute to higher rates of malnutrition, anemia, and micronutrient deficiencies compared to urban counterparts [16]. Moreover, low population density and weak purchasing power in rural communities hamper food logistics and the distribution of perishable goods, leading to higher prices or lower quality, thereby further compromising residents’ nutrition-related health [18, 20, 21].

In response to growing concerns about malnutrition and overweight in rural China, this study aims to investigate the impact of the food environment on nutrition-related health. Drawing on field survey data from Shaanxi Province, this study focuses on how the food environment and its sub-dimensions—namely availability, accessibility, and affordability—affect BMI and the likelihood of being overweight. Beyond assessing direct effects, the study also investigates potential mechanisms, identifying nutrition literacy and dietary quality as mediating pathways through which the food environment influences health. By establishing these causal links and unpacking how food environments shape health behaviors, this research provides evidence-based insights to support the design of targeted public health interventions in rural and resource-constrained settings.

This study makes several key contributions to the literature. First, whereas existing research often relies on a single-dimensional measure of the food environment, we construct a multidimensional index incorporating food availability, accessibility, and affordability. This approach addresses a notable gap in the literature and offers a more comprehensive understanding of the food environment’s impact on nutrition-related health. Second, while prior studies have explored the causal relationship between the food environment and nutrition outcomes, our analysis focuses specifically on rural China and distinguishes between two major types of food outlets—supermarkets and free markets. By investigating whether the food environment can reduce the probability of being overweight, this study offers a more nuanced understanding of its effects on nutrition outcomes. Finally, to our knowledge, this is the first research to jointly incorporate both nutrition literacy and dietary quality as potential mechanisms into the analysis. These findings provide important implications for rural development and public health strategies in developing countries.

## Literature review

A substantial body of literature has examined the relationship between the food environment and nutrition-related health outcomes, consistently demonstrating that changes in the availability and accessibility of food retail outlets significantly influence individuals’ dietary behaviors and health status. The prevailing evidence suggests that improvements in the local food environment—particularly increased access to supermarkets, grocery stores, and free markets—are positively associated with better nutritional outcomes and lower prevalence of diet-related health issues such as obesity and malnutrition [22]. In high- and middle-income countries or regions, as well as among more affluent households, the role of the food environment in shaping dietary quality appears to be especially pronounced [23]. Supermarkets and fresh food markets typically offer a wider selection of affordable and nutritious food items, including fruits and vegetables, thereby facilitating healthier consumption patterns [8, 14, 19]. Empirical studies have shown that the presence of such retail outlets is generally correlated with reduced rates of overweight and obesity [21]. For instance, research finds that older adults residing in areas with limited food store density, poor product freshness, and low food variety are at a heightened risk of malnutrition. Access to healthy food is often operationalized through geographic proximity to full-service food retailers such as supermarkets and supercenters [21, 22]. Furthermore, supermarkets benefit from economies of scale, enabling them to offer lower prices, particularly for non-perishable processed food products, as compared to traditional small-scale retailers [18, 24]. This pricing advantage may further enhance affordability and accessibility, particularly for low- and middle-income consumers, thereby contributing to improved dietary choices and health outcomes.

Conversely, several studies have highlighted certain characteristics of the food environment may contribute to adverse nutrition-related health, particularly a higher prevalence of obesity. In some developed countries, especially outside major urban areas, supermarkets tend to focus primarily on the sale of processed and energy-dense food products [25, 26]. Although improved access to such foods can enhance food security among low-income populations, reliance on processed foods does not necessarily lead to better dietary quality and may exacerbate obesity-related health risks. Convenience stores, which are more common in underserved areas, typically offer a limited variety of products, lower-quality fresh produce, and higher prices compared to supermarkets [27]. Supporting this concern, Courtemanche and Carden find a positive association between the density of Wal-Mart Supercenters and regional obesity rates [28]. Moreover, evidence from quasi-experimental studies suggests that the mere introduction of supermarkets into food-insecure areas does not automatically result in healthier diets or increased consumption of fruits and vegetables [13]. The structure of the local food environment, including the types and distribution of food outlets, plays a crucial role in shaping individuals’ access to nutritious options. Proximity to healthy food retailers is positively associated with higher dietary quality, while residents of “food deserts” or “food swamps”—areas dominated by fast food outlets and convenience stores—tend to exhibit poorer diets and higher rates of obesity [29]. For instance, neighborhoods characterized as food swamps experience significantly higher obesity rates, although this relationship weakens in areas where residents exhibit higher levels of mobility [30]. Similarly, greater exposure to fast food restaurants is associated with increased obesity prevalence [31]. However, the relationship between food retail access and health outcomes is not universally consistent; for example, studies have found no statistically significant association between supermarket shopping and BMI, indicating that contextual factors may mediate the relationship between the food environment and health outcomes [26, 32].

In addition, the potential mechanisms of how the food environment impacts nutrition-related health remain insufficiently investigated. Few studies consider both factors as simultaneous mediating channels through which the food environment affects health—particularly in the context of rural developing regions. Drawing on existing evidence, we propose that two key intermediary mechanisms— nutrition literacy and dietary quality—are particularly relevant in understanding how food environments, especially those structured around supermarkets and free markets, affect health outcomes. Nutrition literacy encompasses individuals’ knowledge, attitudes, and behaviors related to nutrition, including an understanding of nutrient composition, the ability to make informed food choices, and the capacity to manage dietary intake [33]. Supermarkets, restaurants, and food packaging labels serve as important sources of nutritional information, potentially contributing to greater consumer awareness regarding nutrient composition and the associated health outcomes of their dietary intake [34]. A growing body of empirical evidence indicates that individuals with higher education and improved nutrition literacy are positively associated with healthier dietary patterns and a lower prevalence of overweight and obesity. Beyond nutrition literacy, the food environment contributes directly to shaping dietary behaviors and diet quality. For instance, food environments characterized by access to supermarkets are generally associated with higher dietary quality, including greater dietary diversity and adherence to nutritional guidelines [7]. However, this relationship is not uniform across all types of retail formats. While some evidence suggests that supermarkets can promote healthier diets, others indicate that certain large-scale retailers, such as Walmart Supercenters, are linked to reduced diet quality, marked by increased consumption of processed and ultra-processed foods [35]. Nonetheless, expanding access to healthy food options remains critical, as it not only improves diet quality but also contributes to the prevention of nutrition-related chronic diseases [19]. For example, existing literature consistently shows that supermarket patronage is associated with improved dietary quality, including higher scores on measures such as HEI [22, 36].

The competing arguments suggest that the effect of the food environment on nutrition-related health outcomes is complicated and further evidence is needed to understand this linkage. Prior research has disregarded several important aspects. First, prior studies often adopt a fragmented perspective, focusing narrowly on individual components of the food environment—such as availability, accessibility, or affordability—and specific outlet types like supermarkets or fast-food restaurants. These studies frequently emphasize interventions within stores and food purchasing behavior, while overlooking broader, long-term exposure to the built food environment and its direct impact on health outcomes, particularly obesity. Second, empirical research that links objective measures of the food environment with individual health indicators remains limited, especially outside Western contexts. Existing research frequently uses self-reported diet data, which may involve recall and measurement bias, while few studies evaluate tangible nutrition outcomes, such as anthropometric indicators. Moreover, findings from high-income countries are not always transferable to settings like China, where dietary habits, market structures, and urban–rural disparities differ significantly. Third, the mechanisms through which the food environment affects adult health—particularly in rural and low-income settings—have received little attention. In these contexts, food access is often constrained by poor infrastructure and weak market integration. However, evidence of how such rural food environments influence health outcomes is scarce. Addressing these gaps is essential to inform context-specific policies aimed at promoting nutrition and reducing health disparities, particularly in support of the “Healthy China 2030” initiative and the broader nutrition transition.

## Materials and methods

### Sample

This study utilizes data from the field survey in August 2022 in Shaanxi Province. The survey employed a combination of hierarchical sampling and random sampling techniques to select samples. The specific sampling process is as follows: First, five prefecture-level cities—Yan’an, Xianyang, Baoji, Hanzhong, and Ankang—were randomly selected based on the geographical and dietary distinctions across the province. Second, one county was randomly chosen from each city’s ranked population (with two counties selected in Yan’an). Third, towns within each county were categorized into high, medium, and low economic groups based on per capita industrial output and one town was randomly selected from each group. Within each town, one relatively affluent and one relatively poor village were chosen. In each village, 15 rural households were randomly surveyed through face-to-face questionnaires, supplemented by in-depth interviews. As a region encompassing Northern Shaanxi, Guanzhong, and Southern Shaanxi, the province reflects the dietary habits of Northern, Central, and Southern China. Its selection enhances sample representativeness. Moreover, Shaanxi exhibits significant economic disparities and pronounced urban–rural differences, making it an ideal case for analyzing regional variations in nutrition-related health and informing policy decisions.

The data was collected through face-to-face interviews, and the questionnaire mainly includes individual and household characteristics, 24-h food consumption of 2 days, and the local food environment. A total of 540 questionnaires were distributed, of which 520 were valid after excluding those with missing or anomalous data, yielding an effective response rate of 96.30%.

### Variables

#### Nutrition-related health

The primary nutritional outcome variables in this study are BMI and overweight status. BMI is computed as the ratio of weight (kg) to height squared (m^2^). According to established BMI criteria for Chinese adults, nutrition outcomes are divided into four categories: undernutrition is defined by a BMI less than 18.5, normal weight ranges from a BMI of 18.5 up to but not including 24, overweight is indicated by a BMI between 24 and less than 28, and obesity is characterized by a BMI of 28 or higher [37]. Additionally, overweight is analyzed as a binary variable, where individuals with a BMI ≥ 24 are assigned a value of 1, and those with a BMI < 24 are assigned a value of 0.

#### Food environment

The main independent variable employed in this research is the food environment, with a focus on supermarkets and free markets, with a focus on the ease and difficulty of food supply for rural residents at the micro level. It specifically investigates food environment issues related to the physical built environment, while excluding virtual food environments and non-spatial perspectives, such as food heritage and food culture. Drawing on existing research, the analysis incorporates three key dimensions of the food environment: food availability, food accessibility, and food affordability [17, 38–40]. A food environment is constructed using principal component analysis. Specifically, food availability is measured by the density of supermarkets and free markets, calculated as the ratio of the number of these outlets within a 5-km radius of the village to the village population [40, 41]. Food accessibility is assessed by the distance between the village and the nearest supermarkets and free markets [14], while food affordability is evaluated using the ratio of daily per capita household food expenditure to daily per capita income [42].

#### Mechanism variables

Considering the mechanisms through which the food environment may influence the nutrition-related health of rural residents, the indicators to measure nutrition literacy and dietary quality are constructed.

Nutrition literacy scores are based on the nutrition literacy scale used in the survey, which includes 12 nutrition-related health questions [33]. Each item is evaluated on a five-point scale, where responses are coded as 1 for correct, − 1 for incorrect, and 0 for neutral or unsure, yielding a total possible score between 0 and 12.

The CHEI and DBI are employed as proxy measures for evaluating dietary quality in this research, which is calculated using data from residents’ 24-h dietary intake over two days. Given the limitations of existing Chinese dietary indices and the methodological advantages of the CHEI, Yuan et al. established a new CHEI based on the DGC-2016 and validated its effectiveness and reliability [43]. CHEI includes 17 food groups, with 12 beneficial food groups receiving higher scores for higher intake and 5 limited food groups receiving lower scores for higher intake. The score ranges from 0 to 100, with higher scores indicating better dietary quality.

The DBI is a composite measure used to assess dietary quality by capturing both insufficient and excessive intake, based on the Dietary Guidelines for Chinese Residents [36]. The DBI quantifies the degree of deviation from balanced dietary guidelines, focusing on the extent of deviation, and providing a comprehensive basis for diet quality research and nutritional interventions. In this study, the DBI is applied to measure residents’ food consumption quality. Scores are assigned to food groups according to intake recommendations: zero indicates compliance, negative values reflect under-consumption and positive values indicate excess. The total score ranges from 0 to 57, with higher scores representing greater dietary imbalance—categorized as balanced (0), mild (1–11), moderate (12–23), or severe imbalance (24–32). The DBI thus provides a standardized basis for linking dietary patterns to health and economic outcomes.

#### Control variables

Based on health capital demand theory and existing literature [8, 9, 17, 32], this study controls for other factors that influence the nutrition-related health of rural residents, including individual characteristics (age, gender, education level, marital status, alcohol consumption, and labor intensity), household characteristics (household size and income), and village-level characteristics (village area and population size).

### Empirical framework

#### Exploring the food environment and nutrition-related health

Given that the dependent variable, BMI, is continuous, this study employs OLS to analyze the impact of the food environment on the BMI of rural residents. The model is specified as follows:

1 $$Y_{i} = \beta_{0} + \beta_{1} FE_{i} + \beta_{2} X_{i} + \varepsilon_{i}$$

where $Y_{i}$ represents the nutrition-related health indicator BMI, $FE_{i}$ denotes food environment-related indicators, and $X_{i}$ includes individual and household-level control variables. The coefficients $\beta_{0}$, $\beta_{1}$, and $\beta_{2}$ are to be estimated, with $\varepsilon_{i}$ capturing the random disturbance.

Given the increasing prevalence of overweight, this study further investigates the impact of the food environment on the probability of being overweight. Since overweight status is a binary variable, a Probit model is used for estimation. The regression model is specified as:

2 $$\Pr ob\left( {Y_{1i} = 1|X_{i} } \right) = \Phi \left( {\alpha_{1} FE_{i} + \alpha_{2} X_{i} + \omega_{i} } \right)$$

where $Y_{1i}$ is a binary variable indicating whether a rural resident is overweight, $FE_{i}$ represents the food environment, and $X_{i}$ includes individual and household characteristics as control variables. $\alpha_{1}$ and $\alpha_{2}$ are the coefficients of the key explanatory variable and control variables, respectively, while $\omega_{i}$ is the random error term.

If the core variable food environment is exogenous, Eqs. (1) and (2) can be directly estimated. However, the estimation of the above methods may produce biased and inconsistent results due to potential endogeneity issues [40]. First, in addition to the core explanatory and control variables, residents’ nutrition-related health may be influenced by other unobservable factors, leading to omitted variable bias. Second, residents with different levels of nutrition-related health could influence local food retail outlets, significantly increasing or decreasing the supply of certain foods, which could create a bidirectional causality. Third, some regions with a greater focus on nutrition-related health may have residents who are more likely to live near supermarkets or fresh food markets, potentially leading to self-selection bias. To mitigate endogeneity problems and ensure the scientific accuracy of the conclusions, this study re-estimates the impact of the food environment on nutrition-related health using the 2SLS and IV-Probit models. Given the relatively small sample size, we employed nonparametric bootstrap methods with 1,000 replications to obtain more robust standard errors for all models. This approach helps address the limitations of conventional inference in small samples.

In this study, the average value of the food environment at the town level is employed as a potential instrumental variable (IV), excluding each village’s own value [8, 44]. The use of higher-level averages as instruments for lower-level analyses is a well-established approach in the literature. The logic behind this is that while city-level average of the food environment exhibits strong correlations with community-level conditions, they seldom directly impact the nutrition-related health outcomes of individuals residing in communities excluded from the aggregation process. Besides, we employ three IVs to address endogeneity concerns in estimating the effects of the food environment: “highway exits within 10 km of the village”, “number of bus stations in the village”, and “household employment rate” corresponding to food availability, food accessibility, and food affordability, respectively [14, 20, 40]. The number of highway exits serves as a proxy for regional infrastructure development, which influences the availability of food outlets. Since the placement of highway exits is determined by macro-level infrastructure planning, it is unlikely to be directly correlated with the nutrition outcomes of individual rural residents. Similarly, the number of bus stations captures the level of physical access to food markets. As public transportation infrastructure is typically planned based on population mobility needs rather than health considerations, this variable satisfies the exogeneity requirement. Together, these variables reflect the geographic characteristics and transportation conditions of each village. Villages with better transportation infrastructure tend to have improved access to food and other goods produced outside the local area, which facilitates the development of the food environment. The household employment rate influences income and thus food affordability, but once income and expenditure are controlled for, it is not expected to directly affect nutrition-related health outcomes, satisfying the exclusion restriction. In practice, to make sure that all chosen IVs are valid, we conduct estimations to assess their relevance and exogeneity. The results confirm the validity of these instruments, which will be utilized in further analyses.

#### Underlying mechanisms of food environment effects on health

The main goal of this paper is to examine how the food environment contributes to nutrition-related health through specific pathways. Guided by the existing body of research, two primary pathways are analyzed: nutrition literacy and dietary quality. The analysis aims to analyze relationships between food environment and mechanism variables. The model is given as follows:

3 $$M_{it} = \beta_{0} + \beta_{1} FA_{it} + \beta_{2} X_{it} + \varepsilon_{it}$$

In Eq. (3), $M_{it}$ represents indicators examining Nutrition literacy and dietary quality including CHEI and DBI. Control variables are identical to those in the model (1), $\varepsilon_{it}$ is assumed to be normally distributed with standard parameters.

## Empirical results

### Descriptive statistics

Table 1 defines the dependent, independent, and control variables and presents their descriptive statistics. The average BMI of rural residents is 23.379, with an overweight rate of 39.8%, comparable to the national average but with a higher proportion of overweight individuals [9–11]. The food environment in supermarkets and free markets has mean values of 0.221 and 0.207, respectively. Specifically, mean values for food availability are 0.028 (supermarkets) and 0.039 (free markets), while food accessibility, measured by average distance, is 5.224 km and 3.805 km, respectively. Food affordability scores average 7.302 for supermarkets and 6.936 for free markets. For mechanism variables, the average nutrition literacy score is 10.776. Dietary quality is assessed using the DBI averaging 5.640, and the CHEI with a mean of 28.356. Compared to prior studies, rural Shaanxi residents exhibit relatively high nutrition literacy but lower dietary quality [8, 36, 45]. Detailed statistics for control variables are provided in Table 1.

**Table 1:** Descriptive statistics of selected key variables

Variables	Definition	Mean	S.D
*Dependent variables*			
BMI	Body Mass Index (kg/ m^2^)	23.379	5.022
Overweight	1 If BMI ≥ 24 and 0 otherwise	0.398	0.489
*Independent variables*			
Supermarkets-based food environment (FE-S)	Construct a comprehensive food environment index grounded in the dimensions of food availability, accessibility, and affordability within supermarkets	0.221	0.127
Free markets-based food environment (FE-F)	Construct a comprehensive food environment index anchored in the dimensions of food availability, accessibility, and affordability within free markets	0.207	0.126
Supermarkets-based food availability	The density of supermarkets in this village (per 10,000 persons)	0.028	0.031
Free markets-based food availability	The density of free markets in this village (per 10,000 persons)	0.039	0.035
Supermarkets-based food accessibility	The distance (in kilometers) from this village to the nearest supermarket	5.224	3.244
Free markets-based food accessibility	The distance (in kilometers) from this village to the nearest free market	3.805	2.999
Supermarkets-based food affordability	The ratio of per capita daily food expenditure—calculated using supermarket unit prices—to per capita daily income (in CNY)	7.302	15.257
Free markets-based food affordability	The ratio of per capita daily food expenditure to per capita daily income (in CNY)	6.936	13.143
*Mechanism variables*			
Nutrition literacy	Index of nutrition literacy	10.776	1.659
DBI	Diet quality distance of diet balance index	59.159	6.252
CHEI	Chinese Healthy Eating Index	28.356	7.303
*Control variables*			
Gender	1 if male and 0 if female	0.471	0.499
Age	Years of age	56.932	12.391
Marriage	1 if marred and 0 otherwise	0.867	0.339
Education	Highest education completed: 1 = primary, 2 = secondary, 3 = tertiary	1.696	0.548
Drinking	1 if currently drinking and 0 otherwise	0.413	0.493
Heavy activity	1 if heavy activity at work and 0 otherwise	3.377	1.020
Members	Number of household members	2.971	1.368
Income	Per capita household income in OECD standard (thousand CNY^a^)	6.048	4.944
Area	The area of the village (1,000 m^2^)	2.811	2.828
Population	Total population of the village (thousand)	4.589	2.708
N	520		

^a^CNY stands for Chinese yuan; one CNY equals 0.147 US dollars in August 2022

### Benchmark regression results

Baseline regression results in Table 2 indicate that a food environment based on supermarkets and free markets significantly reduces BMI and the probability of being overweight. Empirical results indicate that enhancing the food environment by 1% leads to a significant average decline in BMI, amounting to approximately 35.8% and 63.9%, respectively. Consistent with our estimates, a one standard deviation rise in food environment indicators corresponds to a reduction of 12.1% and 21.0% in the relative probability of being overweight. This finding aligns with previous research [40, 46]. All reported standard errors are bootstrap-based (1000 replications) to improve inference accuracy under small-sample conditions. The key results remain robust across estimation methods and subgroups.

**Table 2:** Impact of food environment on nutrition-related health of rural residents

Variables	(1)	(2)	(3)	(4)
	BMI	Overweight	BMI	Overweight
FE-S	− 0.358**	− 0.121**		
	(0.165)	(0.049)		
FE-F			− 0.639***	− 0.210***
			(0.212)	(0.051)
Gender	− 0.528	− 0.154	− 0.516	− 0.149
	(0.536)	(0.140)	(0.537)	(0.142)
Age	0.106	0.093**	0.065	0.087**
	(0.097)	(0.040)	(0.099)	(0.041)
Age^2^	− 0.001	− 0.001**	− 0.000	− 0.001*
	(0.001)	(0.000)	(0.001)	(0.000)
Marriage	0.633	0.115	0.960	0.236
	(0.624)	(0.199)	(0.601)	(0.198)
Education	0.104	0.090	0.146	0.103
	(0.234)	(0.072)	(0.233)	(0.073)
Heavy activity	0.054	− 0.042	0.076	− 0.040
	(0.228)	(0.062)	(0.225)	(0.062)
Drinking	0.277	0.179	0.131	0.133
	(0.571)	(0.142)	(0.559)	(0.142)
Members	− 0.389**	0.010	− 0.388**	0.013
	(0.180)	(0.049)	(0.179)	(0.048)
Income	0.098**	0.013	0.089*	0.011
	(0.047)	(0.012)	(0.048)	(0.012)
Area	0.114	0.014	0.114	0.014
	(0.067)	(0.024)	(0.067)	(0.024)
Population	− 0.605	0.373	− 0.783	0.334
	(0.661)	(0.242)	(0.648)	(0.246)
N	520	520	520	520

Standard errors are in parentheses; ***p < 0.01; **p < 0.05; *p < 0.1. Bootstrap standard errors in parentheses based on 1000 replications

The impact of food availability, accessibility, and affordability on residents’ nutrition-related health outcomes is presented in Appendix Tables 8 and 9. Estimation results reveal that food availability exerts a statistically significant negative impact on both BMI and the likelihood of being overweight. For food accessibility, a significant positive effect on BMI and overweight prevalence is observed in free markets, whereas no significant relationship is found in the supermarket-based analysis. Regarding food affordability, results indicate a significant negative effect on BMI at the 5% level, but no significant impact on overweight probability in either supermarkets or free markets.

A statistically significant adverse impact of the food environment on adult nutritional status is evident from the estimates. Yet, as previously discussed, possible endogeneity may affect the accuracy of these results, necessitating further robustness verification.

### Correcting for endogeneity bias

From Table 3, the endogeneity test rejects the exogeneity hypothesis at the 1% level, indicating that food environment variables are endogenous. The Cragg–Donald Wald F-statistic is well above Stock-Yogo’s 10% critical value of 16.38, passing the weak instrument test and confirming that the IVs are properly selected with no issues of weak instruments. Results in models (1) and (3) indicate that the coefficients of the first-stage instrumental variables are significantly positive at the 1% level. Based on supermarkets and free markets, results indicate that the food environment has a significant negative impact on BMI at a 5% and 1% level, respectively. One percent increase in food environment based on supermarkets and free markets is associated with an average BMI reduction of 1.034 and 0.773, respectively. Considering that the threshold for overweight is a BMI of 24, a doubling of the food environment significantly reduces the risk of overweight among adults.

**Table 3:** IV estimation of the impact of the food environment on BMI and overweight

Variables	(1)	(2)	(3)	(4)
	BMI	Overweight	BMI	Overweight
FE-S	− 1.034**	− 0.292***		
	(0.449)	(0.104)		
FE-F			− 0.773***	− 0.244***
			(0.315)	(0.082)
Controls^a^	Yes	Yes	Yes	Yes
*First stage:*				
IV	0.964***	0.965***	1.014***	1.014***
	(0.060)	(0.060)	(0.056)	(0.056)
*p*-value	0.000	0.067	0.000	0.058
Cragg–Donald Wald F	122.37		340.90	
AR		7.38***		9.69***
Wald		7.15***		9.82***
N	520	520	520	520

Standard errors are in parentheses; ***p < 0.01; **p < 0.05; *p < 0.1. Bootstrap standard errors in parentheses based on 1000 replications

^a^Controls include all control variables in Table 2

Moreover, the IV Probit estimation results from models (2) and (4) show that the AR and Wald tests are significant at the 1% level, rejecting the null hypothesis and confirming the strength of the instrumental variables. The instruments satisfy the empirical criteria for relevance and exogeneity. The results indicate that the food environment significantly reduces the probability of being overweight at the 1% level. Specifically, a one-standard-deviation increase in the food environment based on supermarkets and free markets decreases the likelihood of being overweight by 29.2% and 24.4%, respectively. These findings suggest that improving the food environment can effectively lower BMI and overweight risk, enhancing nutrition-related health, consistent with previous research [17, 28]. Supermarkets and free markets are generally considered key sources of healthy food. As access to these outlets increases, residents have greater opportunities to purchase nutritious foods, leading to improved dietary intake and, in the long run, a reduction in BMI and overweight prevalence [38].

The impact of each subdimension of the food environment on the BMI and overweight of rural residents is presented in Appendix Tables 10 and 11. The results show that food availability and food accessibility in areas with supermarkets and free markets significantly affect nutritional outcomes. As food accessibility in this study is measured by distance, higher accessibility implies greater distance, reducing convenience and lowering the frequency of healthy food purchases, thereby negatively impacting nutrition-related health. Similar to prior studies, our results emphasize the significant role of food availability and access in curbing the growing overweight problem in rural areas [15]. Greater distances to food outlets limit access to healthy food, increasing the likelihood of being overweight. However, food affordability in both supermarkets and free markets has no significant effect on BMI or overweight probability. One possible explanation is that even when food is economically accessible, individuals may not necessarily make healthier dietary choices due to limited nutrition knowledge, entrenched eating habits, or cultural preferences. Moreover, in rural or low-income settings, improved affordability may lead to increased consumption of inexpensive, calorie-dense, but nutrient-poor foods, thereby offsetting potential health benefits [24].

### Heterogeneity analysis

#### Education heterogeneity

To examine the heterogeneous effects of the food environment on the BMI of rural residents and overweight by different education levels, the sample is divided into two groups: those with at least junior high school education and those with primary school or lower education. In Table 4, models (1) and (2) represent the group with at least junior high school education, while models (3) and (4) represent the group with primary school or lower education.

**Table 4:** The effect of food environment on BMI and overweight by education

Variables	(1)	(2)	(3)	(4)
	Junior high school and above		Primary school and below	
	BMI	Overweight	BMI	Overweight
FE-S	− 0.147	0.056	− 1.629***	− 0.485***
	(0.770)	(0.212)	(0.493)	(0.110)
Controls	Yes	Yes	Yes	Yes
*First stage:*				
IV	0.970***	0.943***	0.987***	0.987***
	(0.164)	(0.164)	(0.106)	(0.106)
Cragg–Donald Wald F	34.93		86.29	
*p*-value	0.000		0.000	0.004
AR		14.23***		14.23***
Wald		12.28**		12.28**
FE-F	− 0.101	− 0.016	− 1.234***	− 0.439***
	(0.521)	(0.131)	(0.333)	(0.115)
Controls^a^	Yes	Yes	Yes	Yes
*First stage:*				
IV	1.122***	1.122***	0.984***	0.984***
	(0.085)	(0.085)	(0.073)	(0.073)
Cragg–Donald Wald F	170.49		179.26	
*p*-value	0.000	0.006	0.000	0.053
AR		9.20***		16.47***
Wald		7.30***		16.00***
N	182	182	338	338

Standard errors are in parentheses; ***p < 0.01; **p < 0.05; *p < 0.1. Bootstrap standard errors in parentheses based on 1000 replications

^a^Controls include all control variables in Table 2

The results in Table 4 indicate that the food environment based on supermarkets and free markets significantly negatively affects BMI and overweight for individuals with lower education levels. This may be explained by the fact that they generally do not possess sufficient knowledge regarding health and nutrition, relying more on the availability and convenience of food rather than making informed choices [34]. Additionally, lower income and education levels make them more sensitive to food prices and accessibility, leading them to select healthier, more affordable food options, which improves their diet. With fewer social support resources, they are more influenced by the food environment in their communities. When healthier food options are available, they are more likely to adjust their eating habits, reducing the intake of unhealthy foods and thus lowering their BMI and overweight rates [30].

#### Gender heterogeneity

To examine potential gender effects of food environment, this study estimates sub-samples for male and female populations. In Table 5, models (1) and (2) represent the male group, while models (3) and (4) represent the female group.

**Table 5:** The effect of food environment on BMI and overweight by gender

Variables	(1)	(2)	(3)	(4)
	Male		Female	
	BMI	Overweight	BMI	Overweight
FE-S	− 2.271***	− 0.524**	− 0.317	− 0.153
	(0.722)	(0.241)	(0.614)	(0.143)
Controls	Yes	Yes	Yes	Yes
*First stage:*				
IV	0.811***	0.811***	1.108***	1.092***
	(0.134)	(0.134)	(0.115)	(0.116)
Cragg–Donald Wald F	36.07		91.20	
*p*-value	0.000		0.000	0.004
AR		8.97**		11.19**
Wald		5.63*		10.99**
FE-F	− 1.586***	− 0.386***	− 0.209	− 0.147
	(0.441)	(0.153)	(0.448)	(0.102)
Controls^a^	Yes	Yes	Yes	Yes
*First stage:*				
IV	0.857***	0.895***	1.136***	1.136***
	(0.081)	(0.081)	(0.075)	(0.075)
*p*-value	0.000	0.018	0.000	0.005
Cragg–Donald Wald F	121.35		225.39	
AR		8.05***		6.93***
Wald		7.97***		6.38**
N	245	245	275	275

Standard errors are in parentheses; ***p < 0.01; **p < 0.05; *p < 0.1. Bootstrap standard errors in parentheses based on 1000 replications

^a^Controls include all control variables in Table 2

The results show that a food environment based on supermarkets and free markets has a significantly negative effect on nutrition-related health for the male group, but it does not significantly affect for female group. This may be because men are often more influenced by the availability and convenience of food, relying on environmental factors such as proximity to food outlets and food prices when making dietary choices [47]. In rural areas, men typically bear the economic responsibilities of the household, making them more likely to take advantage of improved food environments to access healthier food options. Additionally, men generally have lower health awareness compared to women, making them more sensitive to changes in the food environment, leading to adjustments in their dietary habits [5].

#### Age heterogeneity

With the development of urbanization, the average age of rural residents has gradually increased, and the population of left-behind elderly individuals in rural areas has also risen. Therefore, this study mainly focuses on the impact of the food environment on different age groups of rural residents especially for the elderly. In Table 6, models (1) and (2) represent the group aged 60 and above, while models (3) and (4) represent the group under 60. The results show that the food environment, based on supermarkets and free markets, significantly reduces BMI and overweight for the group aged 60 and above, but no significant effect on BMI for another group. This may be primarily because older adults have a limited range of activities and rely more on the local food environment, while their health is more sensitive to nutritional intake [46]. Additionally, their economic capacity and dietary habits are more constrained, making them more affected by changes in the food environment [41]. In contrast, younger individuals are more mobile and can access diverse food sources through migration, online shopping, or external markets, reducing their direct dependence on the local food environment [9].

**Table 6:** The effect of food environment on BMI and overweight by age

Variables	(1)	(2)	(3)	(4)
	Age above 60		Age below 60	
	BMI	Overweight	BMI	Overweight
FE-S	− 1.295**	− 0.349**	− 0.694	− 0.267
	(0.577)	(0.191)	(0.681)	(0.178)
Controls	Yes	Yes	Yes	Yes
*First stage:*				
IV	1.059***	1.039***	0.891***	0.891***
	(0.137)	(0.138)	(0.116)	(0.116)
Cragg–Donald Wald F	59.10		58.65	
*p*-value	0.000		0.000	0.027
AR		6.36**		5.73**
Wald		4.25**		3.22**
FE-F	− 0.916**	− 0.285**	− 0.567	− 0.201*
	(0.350)	(0.132)	(0.505)	(0.116)
Controls^a^	Yes	Yes	Yes	Yes
*First stage:*				
IV	1.075***	1.075***	0.991***	0.991***
	(0.086)	(0.086)	(0.073)	(0.073)
Cragg–Donald Wald F	156.07		180.99	
*p*-value	0.000	0.012	0.000	0.049
AR		6.40***		5.83**
Wald		5.89**		4.61**
N	225	225	295	295

Standard errors are in parentheses; ***p < 0.01; **p < 0.05; *p < 0.1. Bootstrap standard errors in parentheses based on 1000 replications

^a^Controls include all control variables in Table 2

### Robustness test

In order to confirm the robustness of the model’s estimation results, this study investigates the impact of the food environment on the nutrition-related health of rural residents by employing methods such as sample reduction, alternative dependent variables measure, and changes in the identification model.

The sample reduction method is applied by trimming the top and bottom 5% of the income distribution, and the influence between the two groups is estimated. The results in Appendix Table 12 show that the food environment, based on both supermarkets and free markets, has a significant negative impact on residents’ BMI and the probability of being overweight. Additionally, the estimated coefficients are consistent with those from Table 3, indicating that the estimation results are robust.

To observe the impact of the food environment on nutrition-related health, especially on overweight, this study re-estimates the results using a multinomial logit model, as shown in Appendix Table 13. Taking normal BMI as the reference category, model (1) presents the regression results for the underweight group, while model (2) presents the results for the overweight group. The results show that for each unit increase in the food environment based on supermarkets and free markets, the probability of being overweight to those with normal BMI decreases by 15.5% and 33.5%, respectively, with significant effects. This further confirms the robustness of the study’s estimation conclusions.

Considering the multidimensionality of health, this study uses the number of chronic diseases of rural residents as a proxy for their nutrition-related health. The results are re-estimated using the 2SLS model. The results in Appendix Table 14 show that the food environment, based on supermarkets and free markets, has a significant negative effect on the number of chronic diseases, indicating that the model estimation results are robust.

### Mechanism analysis

The previous analysis indicates that improving the food environment has a positive impact on the nutrition-related health of rural residents. To further examine the underlying mechanisms, this study selects residents’ nutritional literacy and dietary quality for further analysis, with the results shown in Table 7.

**Table 7:** The effect of food environment on nutrition literacy and dietary quality

Variables	(1)		(2)		(3)	
	Nutrition literacy		DBI		CHEI	
FE-S	0.565*		− 1.430**		1.356***	
	(0.297)		(0.554)		(0.474)	
FE-F		0.222*		− 0.960**		0.853**
		(0.205)		(0.368)		(0.318)
Controls^a^	Yes	Yes	Yes	Yes	Yes	Yes
*First stage:*						
IV	1.015***	1.015***	1.015***	1.015***	1.015***	1.015***
	(0.054)	(0.054)	(0.054)	(0.054)	(0.054)	(0.054)
Cragg–Donald Wald F	120.16	342.45	120.16	342.45	120.16	342.45
*p*-value	0.000	0.000	0.000	0.000	0.000	0.000
N	520	520	520	520	520	520

Standard errors are in parentheses; ***p < 0.01; **p < 0.05; *p < 0.1. Bootstrap standard errors in parentheses based on 1000 replications

^a^Controls include all control variables in Table 2

According to the estimates from Model (1), the food environment significantly enhances residents’ nutritional literacy, with improvements of 56.5% and 22.2% observed for one standard deviation increases in supermarkets and free markets access, respectively. A favorable food environment not only directly impacts individuals’ nutritional health but also enhances nutrition literacy, which in turn promotes healthy dietary behaviors [38]. By improving access to nutrition information and strengthening individuals’ ability to understand and apply nutritional knowledge, a good food environment encourages healthier food choices, better dietary structure, and the avoidance of unhealthy eating habits, ultimately leading to improved nutritional health [13]. Therefore, optimizing the food environment while enhancing nutrition literacy can further amplify its positive effects on health.

Results of models (2) and (3) show that the food environment, based on the supermarket and free market, positively impacts dietary quality. Specifically, a one-unit improvement in the food environment, based on supermarkets and free markets, results in decreases of 1.430 and 0.960 in DBI, and increase of 1.356 and 0.853 in CHEI, respectively. A well-structured food environment optimizes food supply, increases access to healthy foods, and guides dietary choices through pricing policies and nutrition education, thereby improving CHEI scores and reducing DBI imbalances [14, 48]. A higher-quality dietary pattern enhances nutrient intake, lowers the risk of chronic diseases, and improves health.

## Discussion

China's market-oriented reforms over the past 4 decades have significantly reshaped consumption patterns in rural areas. In particular, farmers’ diets have become increasingly dependent on food market development, including changes in the food environment. In this paper, we use data from a specialized survey conducted in August 2022 across five cities in Shaanxi Province, incorporating both individual- and county-level data, with a total of 520 observations. This study analyzes the causal relationship between the food environment, based on supermarkets and free markets, and the nutrition-related health of rural residents in China. It explores how and through which mechanisms the food environment influences nutrition-related health, considering factors like nutritional literacy and dietary quality. Additionally, this research provides a valuable contribution to the broader discourse on the food environment and its impact on the nutrition-related health of rural individuals.

These findings extend the existing literature on the effects of the food environment on nutrition-related health. The estimation results indicate that the food environment plays a significant role in BMI and the probability of being overweight, highlighting its potential role in combating overweight. In particular, a one standard deviation increase in food environment based on supermarkets and free markets is associated with a lower probability of being overweight by 29.2% and 24.4%, respectively. The findings align with existing research both in China and internationally that explores the link between food environment and nutrition-related health outcomes, and in particular support the conclusion that an improved food environment can help reduce the risk of overweight and nutritional imbalance [17, 38, 46]. From the perspective of food environment subdimensions, food availability in supermarkets and free markets is significantly negatively associated with nutrition-related health among rural residents, whereas food accessibility contributes positively to health improvement. No statistically significant relationship is observed between food affordability and nutrition-related health among rural residents. These results align with evidence from developed nations, where improved food availability and access have been associated with reductions in BMI and lower risks of overweight [15, 27, 48]. From an economic point of view, the results are in line with the expectations of supply and demand theory and behavioral economic theory. With the increased availability and accessibility of healthy food, residents can more easily obtain nutritious food such as fresh vegetables and high-quality protein, and reduce their dependence on high-calorie and low-nutrient food [35, 38]. Such a result echoes patterns observed in international studies examining the relationship between food environments and health outcomes, indicating that optimizing the food supply chain and enhancing the layout of rural markets can effectively improve the nutritional status of residents [46].

The mechanism analysis indicates that the food environment can significantly enhance nutrition-related health by improving rural residents’ nutrition literacy and dietary quality (measured by CHEI and DBI). A supportive food environment—characterized by higher availability and accessibility of nutritious foods—can facilitate exposure to healthier food options, nutritional information, and consumption norms. Over time, this exposure may enhance individuals’ nutrition literacy, enabling them to better understand dietary recommendations, interpret food labels, and make informed food choices, which are essential prerequisites for sustained behavioral change [21, 48]. In addition, an improved food environment can promote higher dietary quality by increasing the availability and affordability of diverse, nutrient-rich foods such as fresh fruits, vegetables, whole grains, and lean proteins. This, in turn, helps residents transition away from energy-dense, nutrient-poor diets that are prevalent in food-insecure rural settings. This study provides a first example for future research on investigating the mechanism of the food environment’s effect on adult nutrition-related health through other channels.

Our findings have important policy implications for improving rural nutrition-related health. First, optimizing the spatial distribution of food retail infrastructure—such as by increasing the number of supermarkets, farmers’ markets, and food supply points, particularly in remote and underserved areas—can significantly improve both food availability and accessibility for rural individuals. Second, improving the dietary quality of rural individuals can be facilitated by strengthening agricultural supply chains to ensure more consistent access to diverse, nutritious food options, thereby addressing the prevalent issue of “food uniformity” in rural markets. For example, policymakers could mandate the inclusion of designated “healthy food sections” in rural supermarkets and implement stricter regulations on the promotion and placement of ultra-processed food products to encourage healthier consumer choices [17, 46]. Third, strengthening nutrition education—particularly targeting males, individuals with lower educational attainment, and adults over the age of 60—can enhance dietary knowledge and promote the adoption of healthier eating behaviors. When integrated with broader policy measures, such interventions can contribute to the development of a more supportive food environment, thereby reducing the risk of being overweight and improving the nutritional well-being of rural individuals.

## Conclusion

This study provides novel evidence that the food environment and its sub-dimensions play a significant role in improving nutrition-related health outcomes among rural residents. Specifically, we find that enhanced food environments—based on supermarkets and free markets—are associated with lower BMI levels and a reduced probability of being overweight, underscoring their potential in addressing rural overweight and obesity. Among the sub-dimensions, food availability in these retail outlets is negatively associated with adverse nutrition outcomes, while food accessibility shows a positive contribution to health improvement. Furthermore, the analysis of underlying mechanisms reveals that the food environment promotes better nutritional health by enhancing nutrition literacy and dietary quality among rural populations. These findings suggest that improving the rural food environment is an effective strategy for advancing nutrition-related health. Policymakers should prioritize targeted interventions to improve the food environment, with a particular focus on enhancing food availability and accessibility through the development of supermarkets and farmers’ markets. At the same time, efforts to promote nutrition literacy and healthier dietary behaviors should be strengthened to support long-term public health goals.

This study provides empirical evidence on the impact of the rural food environment on nutritional health; however, several limitations should be noted. To deepen the understanding of how the food environment shapes nutrition-related outcomes, future research could incorporate behavioral experiments or policy evaluations to examine food choice preferences and assess the effectiveness of specific interventions. Additionally, field surveys collecting detailed data on the availability of healthy food options, frequency of purchases, and the quantity or proportion of food acquired from various outlets would offer more nuanced insights with stronger policy relevance. Furthermore, while this study employed three-day food diaries to assess dietary intake—potentially introducing under- or over-reporting biases—this limitation was partially addressed through a concurrent household-level food inventory. Nevertheless, future studies would benefit from longer-term panel data to capture the dynamic and cumulative effects of the food environment on health outcomes. Due to the relatively small sample size, this may reduce the statistical power, particularly in models relying on maximum likelihood estimation. To mitigate this concern, we employed bootstrap procedures to obtain more reliable standard errors. Nonetheless, future studies with larger and more diverse samples are encouraged to validate our findings.

## Data Availability

No datasets were generated or analysed during the current study.

## Appendix 1

See Tables 8, 9, 10, 11, 12, 13 and 14.

**Table 8:** The impact of food environment sub-dimensional indicators on the BMI of rural residents

Variables	(1)	(2)	(3)	(4)	(5)	(6)
Supermarkets-based food availability	− 0.170**					
	(0.075)					
Free markets-based food availability		− 0.186**				
		(0.082)				
Supermarkets-based food accessibility			0.076			
			(0.079)			
Free markets-based food accessibility				0.232***		
				(0.073)		
Supermarkets-based food affordability					− 0.032**	
					(0.021)	
Free markets-based food affordability						− 0.040**
						(0.020)
Controls^a^	Yes	Yes	Yes	Yes	Yes	Yes
N	520	520	520	520	520	520

Standard errors are in parentheses; ****p* < 0.01; ***p* < 0.05; **p* < 0.1. Bootstrap standard errors in parentheses based on 1000 replications

^a^Controls include all control variables in Table 2

**Table 9:** The impact of food environment sub-dimensional indicators on the probability of being overweight among rural residents

Variables	(1)	(2)	(3)	(4)	(5)	(6)
Supermarkets-based food availability	− 0.061***					
	(0.024)					
Free markets-based food availability		− 0.088***				
		(0.035)				
Supermarkets-based food accessibility			0.025			
			(0.025)			
Free markets-based food accessibility				0.058***		
				(0.019)		
Supermarkets-based food affordability					− 0.004	
					(0.006)	
Free markets-based food affordability						− 0.005
						(0.008)
Controls^a^	Yes	Yes	Yes	Yes	Yes	Yes
N	520	520	520	520	520	520

Standard errors are in parentheses; ****p* < 0.01; ***p* < 0.05; **p* < 0.1. Bootstrap standard errors in parentheses based on 1000 replications

^a^Controls include all control variables in Table 2

**Table 10:** IV estimation of the impact of the food environment sub-dimensional indicators on the BMI

Variables	(1)	(2)	(3)	(4)	(5)	(6)
Supermarkets-based food availability	− 1.317***					
	(0.466)					
Free markets-based food availability		− 0.736*				
		(0.506)				
Supermarkets-based food accessibility			1.629***			
			(0.620)			
Free markets-based food accessibility				0.476*		
				(0.273)		
Supermarkets-based food affordability					0.009	
					(0.162)	
Free markets-based food affordability						0.010
						(0.244)
Controls^a^	Yes	Yes	Yes	Yes	Yes	Yes
First stage:						
IV	0.060***	0.354***	− 0.074***	− 0.223***	− 0.009***	0.026***
	(0.015)	(0.062)	(0.015)	(0.051)	(0.017)	(0.103)
*p*-value	0.000	0.000	0.000	0.000	0.000	0.000
Cragg–Donald Wald F	17.68	31.02	22.86	18.68	17.32	16.94
N	520	520	520	520	520	520

Standard errors are in parentheses; ****p* < 0.01; ***p* < 0.05; **p* < 0.1. Bootstrap standard errors in parentheses based on 1000 replications

^a^Controls include all control variables in Table 2

**Table 11:** IV estimation of the impact of the food environment sub-dimensional indicators on the overweight

Variables	(1)	(2)	(3)	(4)	(5)	(6)
Supermarkets-based food availability	− 0.491**					
	(0.415)					
Free markets-based food availability		− 0.450**				
		(0.267)				
Supermarkets-based food accessibility			0.380***			
			(0.139)			
Free markets-based food accessibility				0.256***		
				(0.098)		
Supermarkets-based food affordability					0.192	
					(0.347)	
Free markets-based food affordability						− 0.018
						(0.021)
Controls^a^	Yes	Yes	Yes	Yes	Yes	Yes
*First stage:*						
IV	− 0.168***	− 0.046***	0.213***	0.079***	− 0.501**	− 0.901***
	(0.056)	(0.015)	(0.054)	(0.017)	(0.017)	(0.018)
*p*-value	0.002	0.010	0.001	0.015	0.019	0.037
AR	11.76***	10.87***	11.58***	10.56**	11.86*	11.96*
Wald	6.00*	6.68**	6.93**	8.20**	8.00*	6.92*
N	520	520	520	520	520	520

Standard errors are in parentheses; ****p* < 0.01; ***p* < 0.05; **p* < 0.1. Bootstrap standard errors in parentheses based on 1000 replications

^a^Controls include all control variables in Table 2

**Table 12:** Robustness test of the effect of food environment on nutrition-related health

Variables	(1)	(2)	(1)	(2)
	BMI	Overweight	BMI	Overweight
FE-S	− 1.022**	− 0.301***		
	(0.425)	(0.121)		
FE-F			− 0.758***	− 0.244***
			(0.316)	(0.083)
Controls^a^	Yes	Yes	Yes	Yes
*First stage:*				
IV	0.961***	0.952***	1.012***	1.012***
	(0.087)	(0.087)	(0.054)	(0.054)
Cragg–Donald Wald F	121.93		339.82	
*p*-value	0.000	0.023	0.000	0.005
AR		11.78***		9.68***
Wald		7.51***		9.82***
N	520	520	520	520

Standard errors are in parentheses; ****p* < 0.01; ***p* < 0.05; **p* < 0.1. Bootstrap standard errors in parentheses based on 1000 replications

^a^Controls include all control variables in Table 2

**Table 13:** Robustness test of the effect of food environment on nutrition-related health

Variables	(1)		(2)		
	Underweight		Overweight		
FE-S	0.035		− 0.158*		
	(0.126)		(0.082)		
FE-F		0.010			− 0.338***
		(0.125)			(0.086)
Controls^a^		Yes	Yes		Yes
Log likelihood	− 460.01			− 453.56	
LR statistic	62.26***			61.58***	
N	520	520	520		520

Standard errors are in parentheses; ****p* < 0.01; ***p* < 0.05; **p* < 0.1. Bootstrap standard errors in parentheses based on 1000 replications

^a^Controls include all control variables in Table 2

**Table 14:** Robustness test of the effect of food environment on nutrition-related health

Variables	(1)	(2)
	Disease	Disease
FE-S	− 0.237**	
	(0.035)	
FE-F		− 0.173***
		(0.024)
Controls^a^	Yes	Yes
*First stage:*		
IV	0.956***	1.015***
	(0.089)	(0.057)
Cragg–Donald Wald F	120.16	341.39
*p*-value	0.000	0.000
N	520	520

Standard errors are in parentheses; ****p* < 0.01; ***p* < 0.05; **p* < 0.1. Bootstrap standard errors in parentheses based on 1000 replications

^a^Controls include all control variables in Table 2

## Abbreviations

BMI: Body mass index
CHEI: Chinese healthy eating index
DBI: Diet balance index
IV: Instrumental variable
